# The effects of implementing phenomenology in a deep neural network

**DOI:** 10.1016/j.heliyon.2021.e07246

**Published:** 2021-06-08

**Authors:** Joshua Bensemann, Michael Witbrock

**Affiliations:** School of Computer Science, University of Auckland, Auckland, New Zealand

**Keywords:** Synthetic phenomenology, Neural network, Hebbian learning

## Abstract

There have been several recent attempts at using Artificial Intelligence systems to model aspects of consciousness (Gamez, 2008; Reggia, 2013). Deep Neural Networks have been given additional functionality in the present attempt, allowing them to emulate phenological aspects of consciousness by self-generating information representing multi-modal inputs as either sounds or images. We added these functions to determine whether knowledge of the input's modality aids the networks' learning. In some cases, these representations caused the model to be more accurate after training and for less training to be required for the model to reach its highest accuracy scores.

## 1 Introduction

Discussions about the properties of consciousness have taken place within multiple disciplines. Interested parties include philosophers (e.g. Block and Dennett, 1993; Block, 1995; Chalmers, 1995), neuroscientists (e.g. Crick and Koch, 1990; Hohwy and Frith, 2004), cognitive psychologists (e.g. Dehaene, 2014), and computer scientists (e.g. Bengio, 2017; Reggia and Davis, 2020). The last group listed are mostly artificial intelligence (AI) researchers interested in the related concept of artificial consciousness (see Gamez, 2008; Reggia, 2013). However, attempting to create a conscious AI is not the only way this group can contribute to the discussion. By recreating properties of consciousness within AI models, those researchers can test those properties' effects directly. This is the approach that has been used in this article and has been used by others previously (Arrabales et al., 2010b; Zaadnoordijk and Besold, 2018; Schartner and Timmermann, 2020). This article aims to contribute to the existing literature by introducing experiments using Deep Neural Networks (DNN) trained to classify multi-modal data. We found that it is possible to extract simple representations of the modality being processed by the DNN using information from the hidden layers within that network. Following that, we used these simple representations to aid other DNNs to classify the same multi-modal data. The purpose of these experiments was to determine whether the addition of these representations improved network performance. In our view, the simple representations of modality are analogous to phenomenology awareness of that modality. Therefore, the experiment investigates whether there is any utility in having some limited phenomenology.

### 1.1 Background

The interdisciplinary nature of consciousness studies is a double-edged sword. On the one hand, the field has engaged minds from across the academic community. On the other hand, those minds disagree on foundational concepts, such as what counts as consciousness. For example, some believe that anything – be it biological, artificial, physical, or virtual – that possesses specific architectural components could be conscious (Aleksander and Dunmall, 2003; Aleksander & Morton, 2007, 2008; Arrabales et al., 2010a; Tononi and Koch, 2015). Others believe that specific behaviours are required; if something possesses functions equivalent to consciousness, it is conscious. Some researchers use this functional definition when describing robots demonstrating behaviour such as mirror self-recognition (Takeno et al., 2005) and other self-awareness research (Chella et al., 2020). Defining consciousness architecturally or functionally differs from the neuroscience approach of focusing on biological consciousness by examining the functioning of structures within the (typically human) brain (see Dehaene, 2014 for review).

We approached consciousness from AI's perspective for our experiments, focusing on functional properties rather than physiological mechanisms. Specifically, we have focused on the knowledge representation functions of consciousness. In preliminary experiments, we have referred to this representation as a limited form of phenomenal consciousness (see Bensemann and Witbrock, 2020) because those data provided information about the “senses” required to process the task. However, without a consensus about how to scientifically study phenomenology (see Chan and Latham, 2019 for review), the success of those arguments likely depends on the reader's point of view. For now, we will continue with that point of view. Those opposed to that characterisation can view this as an experiment using internal representations of knowledge to improve an AI system's performance.

Our experiments were primarily motivated by the fact that one electrochemical signal in the brain looks like every other. While it is possible to determine what processes are occurring using signal averaging (see Dehaene and Changeux, 2011), the signal itself is comprised of electrochemical signals that are unreadable to those external to the brain. The existence of subjective cognitive phenomena shows that the brain's hardware must have some mechanism for decoding those signals. In the present study, we were interested in examining an analogist principle in artificial neural networks, where the signals are the values being propagated throughout the network. It is generally difficult, if even possible, to decode that signal within the hidden layers of a DNN by merely examining the values. Due to this difficulty, the first part of our investigation was to determine whether it was possible to partially decode the signals passing through a basic DNN model learning a multi-modal task. In this case, the decoder was a simple Hebbian learner (Hebb, 1949) trained to predict input data's modality.

The second part of our investigation was to determine whether the decoded information could be of any use to DNNs. Our decoding method created a 1-bit representation of the file inputs modality, and we used the decoded information as meta-data input for a hidden layer of a second DNN. The second network was then trained on the original multi-modal classification task to see if the additional information improved the DNN's performance. We believe that representations of modality are sufficient to represent a limited form of phenomenology because human visual, auditory, tactile, olfactory, and other experiences represent the type of input that the brain is receiving from the body. For example, visual experiences represent electromagnetic waves, and auditory experiences are representations of physical waves.

The ability to have experiences caused by the physical world is known as phenomenal consciousness (P-Consciousness; Block, 1995), which is related to yet separate from access consciousness (A-Consciousness; Block, 1995). If a signal's representation is in a state of A-Consciousness, it can be manipulated by the intelligent agent containing that representation. This state occurs when the representation's content is available for higher-order thought and can be reasoned over and used to determine the agent's actions, such as movement and speech. The critical difference between A-Consciousness and P-Consciousness is that the former is manipulating information without any experience of that information. In contrast, the latter is the phenomenological experience of that information. For example, both humans and neural networks can identify images, but only humans will visually experience (i.e. see) those images. This experience is P-Consciousness. The human versus neural network example leads to an essential question about the phenomenological experience: How does the ability to experience an image benefit the experiencer?

Neural network performance in image classification tasks is continuously improving. For example, ImageNet (Krizhevsky et al., 2012) accuracy has increased from 50.9% to 88.5% (Touvron et al., 2020) in less than a decade. We might not know whether P-Consciousness improves image classification performance, but as neural networks approach 100% accuracy, it becomes increasingly likely that phenomenology is unnecessary to perform that task. The same reasoning could be generalised to other sensory modalities (sound, smell, touch, etcetera), which means that the human brain's ability to decode signals into experiences is not required for artificial neural networks to complete classification tasks.

Even if neural networks never reach human performance, there is evidence from human studies that suggest phenomenology is unnecessary for classification tasks. Studies of blindsight patients show that humans can make visual judgements without the experience of sight (Weiskrantz, 1998). Blindsight patients suffer from damage to their primary visual cortex and report being blind even though their eyes and optic pathways are intact. Despite the lack of visual experience, they can perform basic visual identification tasks above chance accuracy (Weiskrantz et al., 1974; Overgaard, 2011). Performing about chance accuracy is evidence that experience is unnecessary for humans to complete some visual tasks. Furthermore, there is evidence that similar conditions exist in other sensory modalities (see Garde and Cowey, 2000; Zucco et al., 2015). The human brain's ability to decode signals into experiences might not even be required for the human brain to complete classification tasks.

The utility of phenomenology has long been the subject of discussion. Some have speculated that it exists to create stability within cognitive processes (Ramachandran and Hirstein, 1997); having a common representation of colour perception allows the representation to be reused in the future. Others suggest that we use the vividness of experience to distinguish between information occurring in real-time, such as images captured by the eyes, compared to mental images of prior inputs (Gregory, 1998). Experimental evidence has suggested that this experience could have some predictive function; our experience of being in control of our body disappears when there is a contrast between what we intend to do with our body and the feedback we receive (Hohwy and Frith, 2004). Alternatively, others have speculated that experience has no purpose and that its existence is a side effect of cognitive processing (Dennett, 2016).

By necessity, discussions on phenomenology's utility are predicated on indirect evidence as it is difficult to manipulate the presence/absence of experience experimentally. With that said, it is possible to induce blindsight using transcranial magnetic stimulation temporarily (TMS; see Ro et al., 2004). However, TMS methods cause transient lesions to the brain, which may disrupt more than just experience. An alternative to TMS is to use AI models as a substitute for the brain. By adding phenomenological properties to existing AI architectures, it is possible to observe whether the resulting models benefit from these additions. These additions provide a stepping stone to identifying the utility of such properties in conscious beings. This approach, known as Synthetic phenomenology (Chrisley, 2009), has been discussed and implemented by others (Arrabales et al., 2010b, 2011; Zaadnoordijk and Besold, 2018), and we hope to add to this discussion by simple experimentation with deep neural networks.

### 1.2 Present experiment

**Figure 1:**
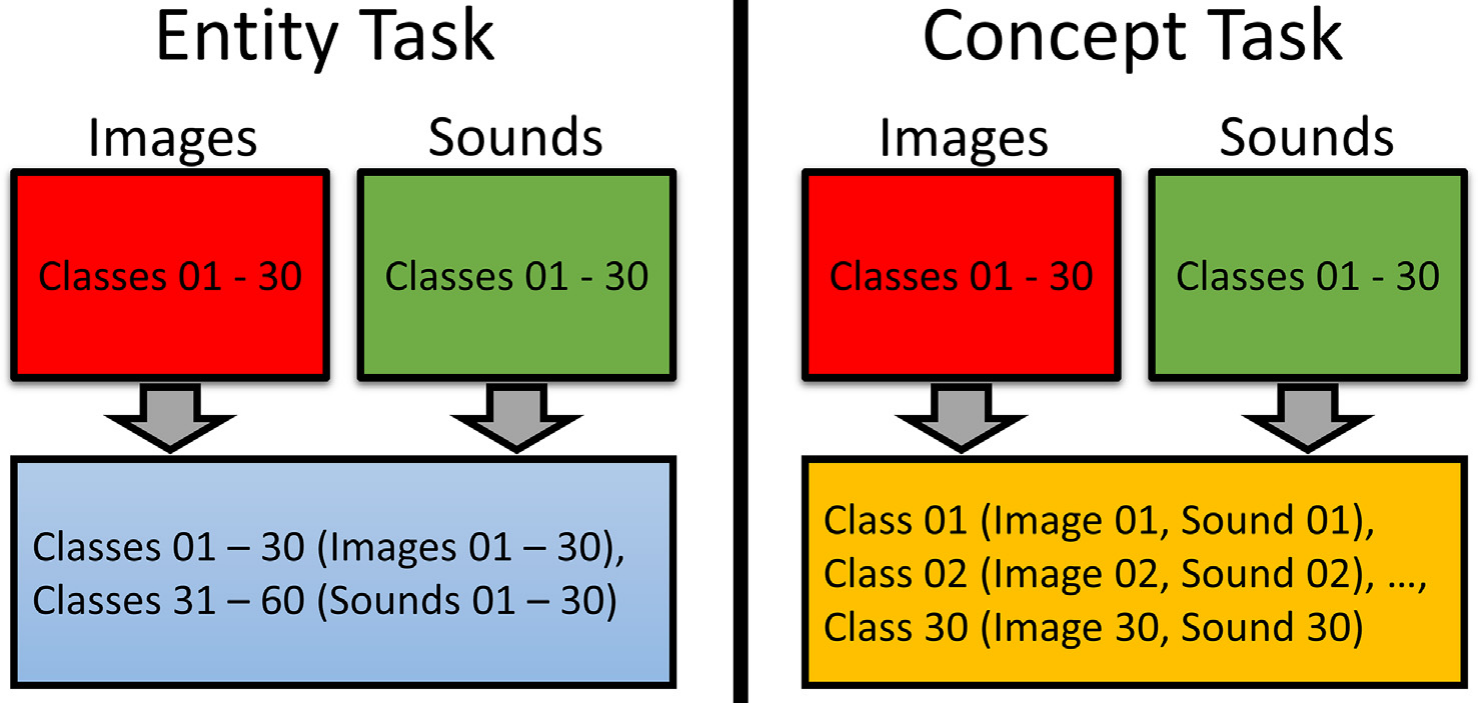
Tasks: A diagram of the Entity and Concept Tasks. Entity task: the models were required to classify 60 different classes consisting of 30 sound and 30 image classes. Concept task: each of the 30 image classes was paired with a sound class to create 30 classes.

There were two general steps to our experiment. First, we trained neural networks to classify both sound and image inputs on two different tasks using the same dataset (see Figure 1). In the Entity task, we trained the models to classify 60 different classes (i.e. entities) consisting of 30 sound and 30 image classes. In the Concept task, each of the 30 image classes was paired with a sound class to create 30 concepts to classify. Without explicitly training the network to differentiate between the sound and image file inputs, we could recover whether the input was initially a sound or an image using the information present in the network's hidden layer. We believe that this approximates an emergent symbol of experience for the processing occurring within the network. Second, we trained networks using both the original inputs for the task and the recovered “experiential” information.

We have termed the models in the second experimental step as “Experimental” to help the reader identify which models have this additional information. In terms of phenomenology, this experiment was to see whether the ability to identify an otherwise indistinguishable signal as either sight or sound impacts classification performance. By providing this ability, we were able to improve the performance of the network in some situations.

The present experiment is an expansion of our preliminary work (Bensemann and Witbrock, 2020). Those results suggested that it was possible to improve a DNNs performance using internal representations of the data modality. However, due to its preliminary nature, that work lacks control conditions included in the present experiment. The preliminary work balanced the number of image and sound examples within the dataset; the present experiment took the additional step of balancing the number of classes.

## 2 Method

### 2.1 Data

The full dataset consisted of two parts, an image dataset and a sound dataset. The image data consisted of the balanced version of the EMNIST dataset (Cohen et al., 2017). Although this set contains 47 classes of handwritten letters and digits, we used only the first 30 classes; this differs from preliminary work, where all 47 classes were used (Bensemann and Witbrock, 2020). The sound data were drawn from the Speech Commands dataset (Warden, 2017), consisting of 30 classes of spoken words.

The sound data were initially in the wave file format and required several pre-processing steps to create an input format compatible with the image data. Each sound was converted into a Mel spectrogram using a sampling rate of 22050 samples per second. The conversion resulted in 128 by 44 matrix representations of each wave file. These representations were then modified to match the 28 by 28 file size of the EMNIST dataset. We achieved this final format by zero-padding the files to a size of 140 by 56. Max pooling was then applied using a pool size of 5 by 2, which resulted in the final format. After pre-processing, we reserved 20% of the sound data for validation.

We normalised the values of both the image and sound datasets to between 0 and 1. The datasets were then combined to make two variations of a final dataset; One version where all 60 classes were separate (Entity Dataset) and one version where each image was paired with a sound to create 30 classes (Concept Dataset).

### 2.2 Models

We created two different baseline models, one for each of the datasets. Both baselines model consisted of 3 convolution layers followed by two dense hidden layers and a dense output layer. Hyperband optimisation (Li et al., 2018) was used to determine each layer's best hyperparameters. This optimisation was run on each of the datasets separately, resulting in one model optimised for the Entity Dataset (Entity Base) and the other optimised for the Concept Dataset (Concept Base). Table 1 shows the complete structure of each model.

**Table 1:** Model Architectures. The parameters selected for each layer of the neural networks after hyperband optimisation.

Model	Both Models	Entity		Concept	
		Filters/Nodes	Activation	Filters/Nodes	Activation
Convolutional Layer 1	3 × 3 Kernel size	32 Filters	Relu	16 Filters	Tanh
Max Pooling Layer 1	2 × 2 Pool Size				
Convolutional Layer 2	3 × 3 Kernel size	64 Filters	Tanh	16 Filters	Relu
Max Pooling Layer 2	2 × 2 Pool Size				
Convolutional Layer 3	3 × 3 Kernel size	16 Filters	Sigmoid	32 Filters	Relu
Max Pooling Layer 3	2 × 2 Pool Size				
Dense Layer 1		512 Nodes	Relu	320 Nodes	Relu
Dense Layer 2		288 Nodes	Tanh	448 Nodes	Sigmoid
Output	Softmax Activation				

A Hebbian learner (Hebb, 1949) was attached to each of the baseline model's dense hidden layers to determine whether the models were developing “representations” of input type. These learners received non-activated outputs from their layer. The outputs were divided by their absolute value, converting all output values into 1 or -1. We trained the Hebbian learners using the output data and a 1 or -1 label representing whether the layer's output was generated via a sound or image file, respectively.

We derived two experimental models from the baseline models (Entity Exp and Concept Exp). Both experimental models had the same structure as the corresponding baseline model. The difference being that we concatenated the output from a Hebbian learner of a pre-trained baseline model on to the data entering the first dense layer of the experimental models. This concatenation provided the experimental models with the equivalent of one bit of additional information during training.

Finally, we developed two control models for Condition 1, Expanded Base and Constant Input. The Expanded Base was created by adding a node to the first dense layer to increase the number of trainable parameters. The Constant Input model was identical to the Exp Model but received a constant input of 1 instead of 1 or -1 from the Hebbian learners. The constant input caused the model to have an equal number of parameters to the Exp Model.

### 2.3 Training

We used the Adam optimiser (Kingma and Ba, 2015) with a learning rate of 0.0005 to train the models. Each model was trained a total of 30 times using the sparse categorical cross-entropy loss function. Each training run lasted for 500 epochs consisting of 1000 steps. A batch of 128 training examples was created by sampling uniformly between the image and sound samples during each step.

Hebbian learners attached to the base models were reset at the start of each epoch and retrained over the 1000 steps. Accuracy data from the learners were collected but otherwise ignored during the baseline model training procedure.

Training experimental models started by pre-training a baseline model until at least one of the attached Hebbian learners achieved greater than 99% accuracy on the validation dataset. Once this occurred, we froze the baseline model weights along with the more accurate of the two Hebbian learners. This process often required one epoch of pre-training. After pre-training, training the experimental model proceeded like baseline training. The exception is that we first passed the batch of data through the pre-trained baseline model and Hebbian learner. The batch was then used as input for the experimental model. Finally, the output from the Hebbian learner was concatenated onto the flattened outputs of the convolutional layers within the experimental model.

We ran four different conditions, each using a different combination of models and dataset. Table 2 shows the complete list of conditions. In Condition 4, neither of the Hebbian learners reached the 99% accuracy criterion during baseline training. Due to this, the learners from a pre-trained Concept Base model were used to provide the additional data required to train the Entity Exp model in this condition.

**Table 2:** Condition Order. The order of conditions run in the experiment.

Condition	Dataset	Models
1	Entity	Entity Base, Entity Exp, Expanded Base, Constant Input
2	Concept	Concept Base, Concept Exp
3	Entity	Concept Base, Concept Exp
4	Concept	Entity Base, Entity Exp

### 2.4 Statistical analysis

We conducted non-parametric tests during the analysis: The Kruskal-Wallis Test (Kruskal and Wallis, 1952), the Conover test with Holms-Method correction (Holm, 1978; Conover and Iman, 1979), and the Mann-Whitney-U (Mann and Whitney, 1947). We chose non-parametric methods to avoid making assumptions about the underlying structure of data.

## 3 Results

### 3.1 Successfully using Hebbian Learners to identify training sample origin

**Figure 2:**
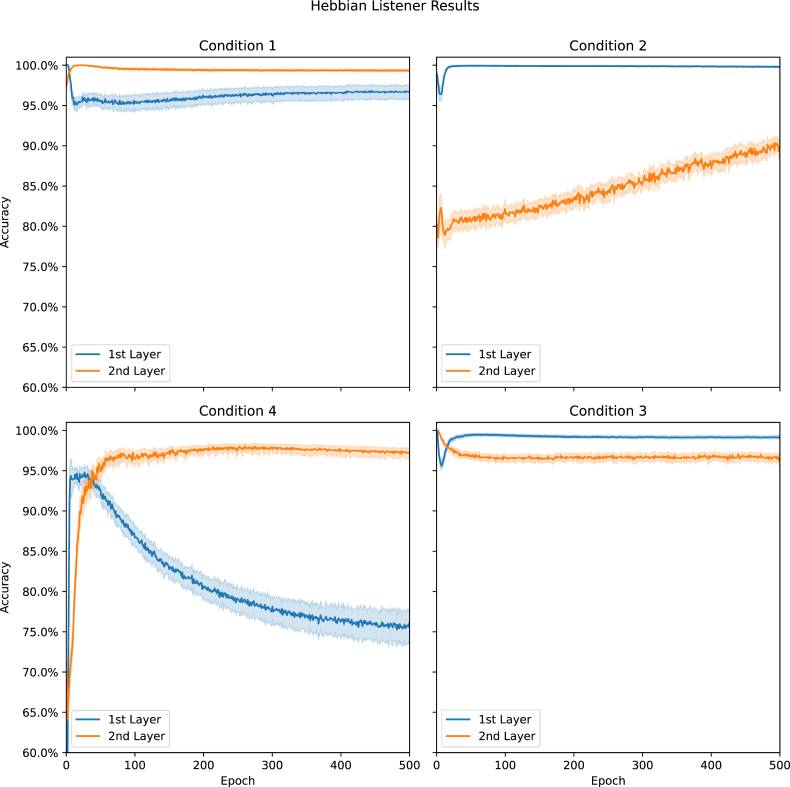
Hebbian Learner Results: The average accuracy obtained from the Hebbian learners attached to the 1^st^ and 2^nd^ dense layers during Baseline Model training for each condition. Data are presented with 95% confidence intervals.

The baseline version of each neural network had a Hebbian learner (Hebb, 1949) attached to both of its dense hidden layers. We trained these learners to identify whether the network's input was sampled from the image or sound datasets using the hidden layer's non-activated outputs. Figure 2 shows the average accuracy of each learner at the end of each epoch. Note, we reset the learners at the start of every epoch; changes in accuracy over epochs indicate how difficult it was to train a learner using the linear outputs during that epoch. The difficulty changes resulted from the modifications made to hidden layers weights by training the neural network; this training was not affected by the Hebbian classifiers.

We obtained accuracies greater than 99% from at least one learner in the baseline models of the first three conditions. Often, only one epoch of training was required to obtain these high levels of accuracy. Focusing on Condition 4, we can see that the 2^nd^ layer learner did exceed 95% accuracy after sufficient training. Interestingly, the 1^st^ layer learner in the same condition was the only one that continued to decrease in accuracy across epochs.

Comparing across conditions (Figure 2), we can see that the Hebbian learner on the 2^nd^ layer was more accurate when training the Baseline Model optimised for identifying all 60 classes (Entity Base). In contrast, the 1^st^ layer learner was more accurate when training the Baseline Model optimised for 30 classes (Concept Base). It also appears that training with the 60 class dataset (Entity Dataset) caused both learners to exceed 95% accuracy regardless of which model was used.

### 3.2 Identifying sample origin improves optimised models' performance

**Figure 3:**
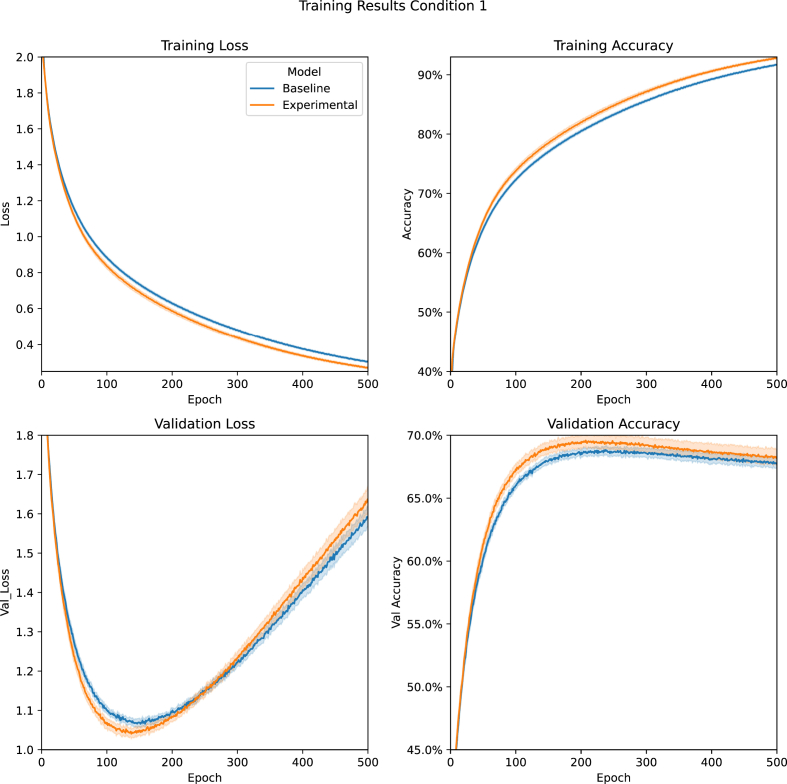
Condition 1 Training Results: A comparision between the Baseline and Experimental Models' average training loss, training accuracy, validation loss, and validation accuracy during Condition 1 (Entity task with optimised models). Data are presented with 95% confidence intervals. See also S1 and S2.

Condition 1 compared performance between four variations of the model optimised for learning the Entity Dataset where the 30 sound classes and the 30 image classes were 60 separate classes. These variations included the Baseline and Experimental Models used in all other conditions, plus the two control models, Constant Input (Experimental Model that always concatenates 1 instead of -1 or 1) and Expanded Baseline (Baseline Model with additional node on the first dense layer). Figure 3 shows the Baseline and Experimental models' average performance; the control models' performances were almost identical to the Baseline, so they were excluded for visual clarity (the entire figure is available in the supplementary materials). The average training loss was lower for the Experimental Model, which resulted in higher average training accuracy. The same pattern was observed in the validation data until training exceeded approximately 150 epochs, at which point the models began to overfit the training data.

**Figure 4:**
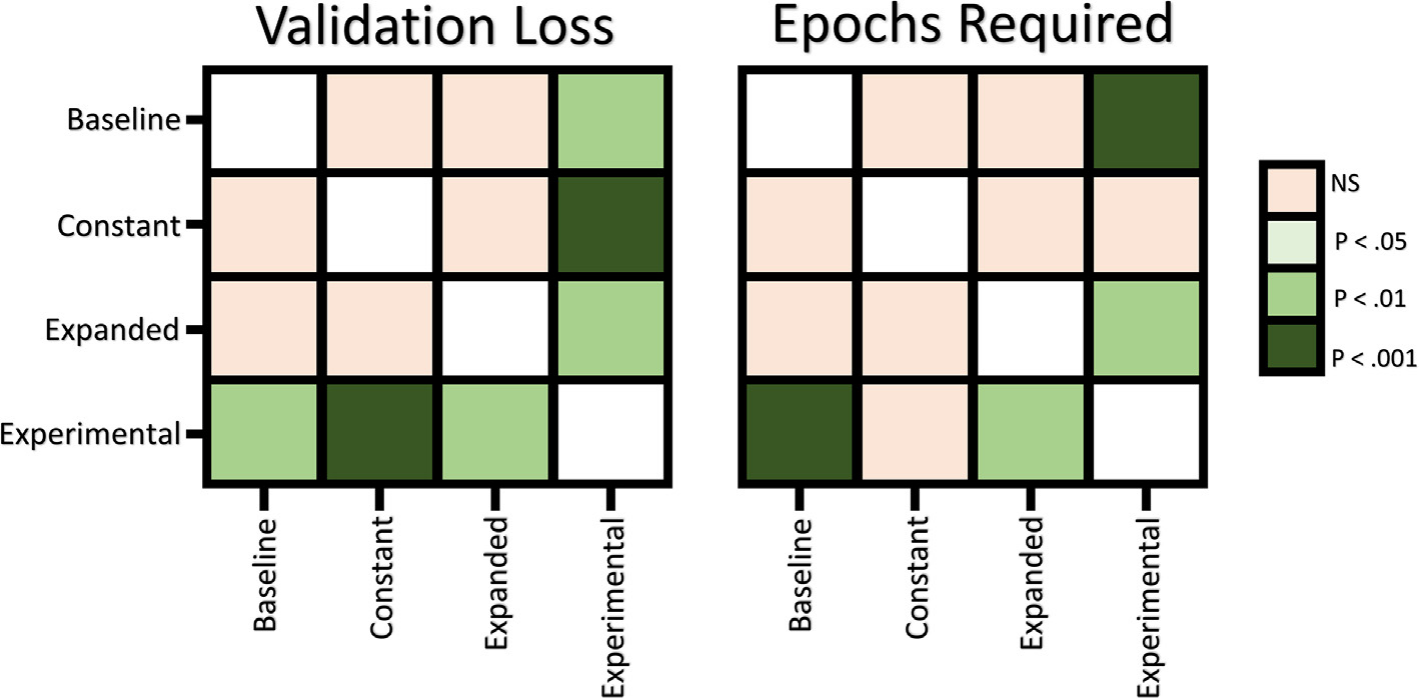
Condition 1 Statistical Test Results: The Conover test results with Holms-Method correction in Condition 1.

The minimum validation loss obtained during each training run of all four models was collected and compared using the Kruskal-Wallis Test (Kruskal and Wallis, 1952). The test confirmed a statistically significant difference between at least one group (*P* < .001). We conducted Post hoc tests using the Conover test with Holms-Method (Holm, 1978; Conover and Iman, 1979) for corrections; results indicate that the Experimental Model significantly outperformed all other models in classification accuracy (Figure 4). We also performed tests on the number of epochs required to reach minimum validation loss. Results indicated a significant difference between groups (*P* < .001), and posthoc tests indicated differences between the Experimental Model and both the Baseline and Expanded Baseline Models; tests indicate there was no difference between the Experimental Model and the Constant Input Model (Figure 4). These results suggest that the Experimental Model achieved maximal performance with less training than the Baseline and Expanded Baseline Models.

Condition 2 trained models on the Concept Dataset, where we paired each of the 30 sound classes with one of the 30 image classes. The models used were optimised to work with this version of the dataset. Due to resource constraints, we could only train the Baseline and Experimental variations of the model.

**Figure 5:**
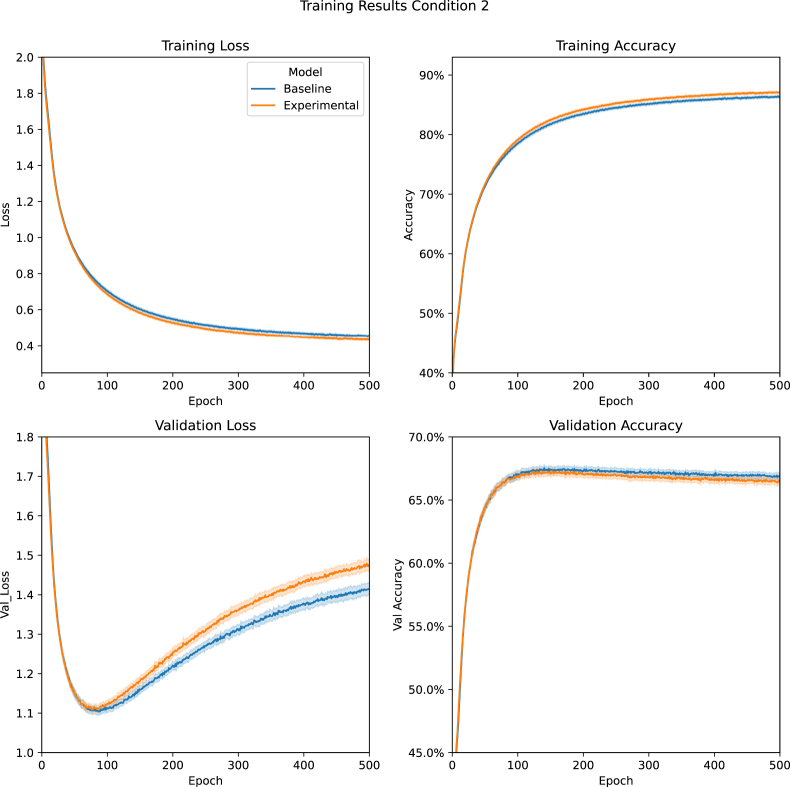
Condition 2 Training Results: A comparision between the Baseline and Experimental Models' average training loss, training accuracy, validation loss, and validation accuracy during Condition 2 (Concept task with optimised models). Data are presented with 95% confidence intervals.

Figure 5 shows the average training results from Condition 2. Mann-Whitney-U (Mann and Whitney, 1947) tests indicated that the Experimental Model's performance in the classification task was equal to that of the Baseline Models (*P* = .31) but that less training was required for the Experimental Model to reach optimal performance (P = .01).

### 3.3 Non-optimised models not improved by identifying sample origin

Conditions 3 and 4 were trained with the Entity and Concept Datasets, respectively. In these conditions, we switched the models used in Conditions 1 and 2 so that each dataset was trained with models optimised for the other dataset. Due to the same resource constraints as Condition 2, we only tested the Baseline and Experimental variations of each model.

**Figure 6:**
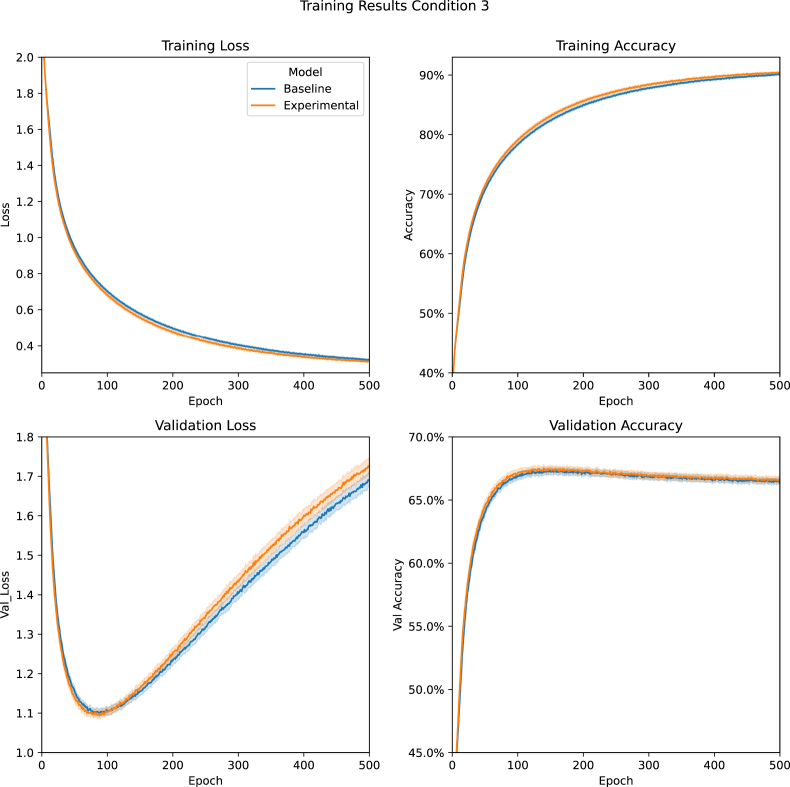
Condition 3 Training Results: A comparision between the Baseline and Experimental Models' average training loss, training accuracy, validation loss, and validation accuracy during Condition 3 (Entity task with non-optimised models). Data are presented with 95% confidence intervals.

Figure 6 shows the results of training the 60 class Entity dataset with the non-optimised model (Condition 3). Mann-Whitney U tests indicated non-significant differences between the models' minimum validation loss (*P* = .23) and epochs required to reach that minimum loss (*P* = .17).

**Figure 7:**
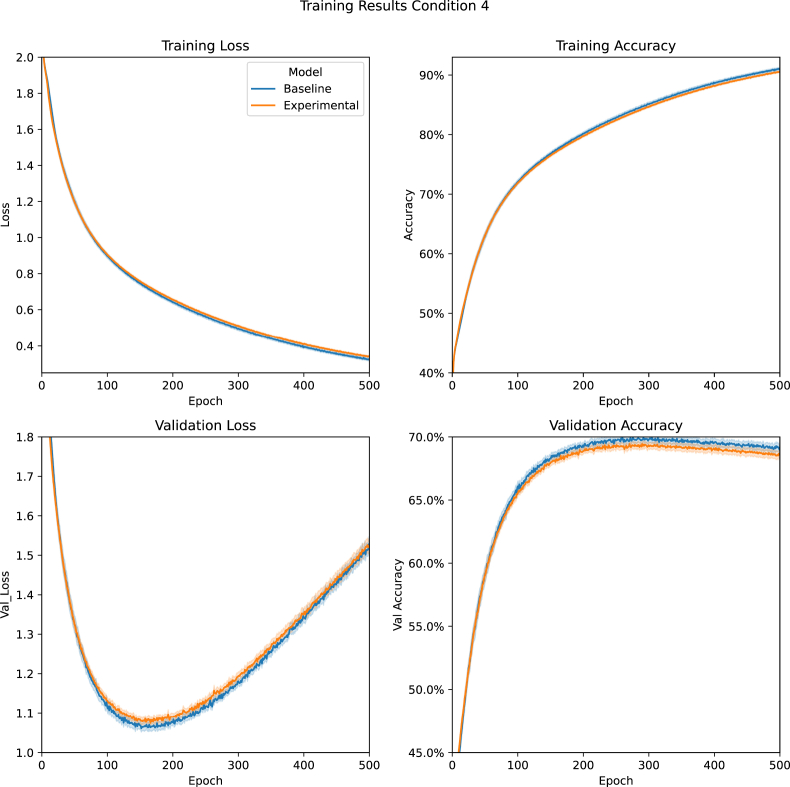
Condition 4 Training Results: A comparision between the Baseline and Experimental Models' average training loss, training accuracy, validation loss, and validation accuracy during Condition 4 (Concept task with non-optimised models). Data are presented with 95% confidence intervals.

Figure 7 shows the results from Condition 4. While the Mann-Whitney U tests indicated significant differences between the minimum validation loss (*P* = .01), this difference was due to the baseline model outperforming the Experimental Model. There was, however, no difference in the number of epochs required to reach minimum validation loss (*P* = .23).

It should also be noted that the minimum validation loss in Condition 4 is lower than that of Condition 2, despite models in Condition 2 being optimised for the Concept Dataset. The optimisation procedure can explain this. The Hyperband optimisation (Li et al., 2018) was applied to the first 50 epochs of training to find models that achieve lower minimum validation loss after 50 epochs. Closer inspection of the data indicates that after 50 epochs, the model from Condition 2 does have a lower validation loss and was chosen by the optimisation procedure.

## 4 Discussion

### 4.1 Decoding signals

The purpose of the Hebbian learners (Hebb, 1949) in the experimental system was to determine whether information about the type of the input, contained implicitly in the hidden layers of a deep neural network, could provide additional information that could aid in learning the input's label. We trained the Baseline Models to identify the 60 or 30 classes within the Entity or Concept Datasets. We did not directly train those models to identify whether each class came from the image or sound dataset. Instead, we trained the Hebbian learners to classify whether an input was an image or a sound by using the hidden layers' linear outputs, and these outputs were changing during training. Being able to classify the hidden layer outputs accurately suggests some organisation occurring during the training of those layers, making it possible to separate the data (see also Veldhoen et al., 2016). Visual inspection of Figure 2 suggests that this organisation changes during training as the learners' accuracy tended to change continually.

Visual inspection of Figure 2 shows that, in general, it was possible to identify which sample each of the inputs came from using the otherwise indistinguishable signal of a hidden layer's linear outputs. This finding was critical to the experiment as we held the assumption that there is some mechanism in the brain that can achieve the same result and that there may be some utility in the ability to do so. We could have emulated this same effect via directly inputting a sound/image label into the Experimental Models; however, showing the process occurring increases the likelihood that the assumption is valid. Furthermore, the Hebbian learners used to make this distinction use Hebb (1949) simple yet biologically plausible learning method. If the brain's ability to distinguish signals is not hardwired at birth, our success with Hebbian learners suggests strongly that this ability could be readily learned.

### 4.2 Baseline versus experimental models

The results from all four experiment conditions provide mixed evidence for whether adding one bit of information to a model to emulate basic phenomenology benefitted the model. The first two conditions provided evidence supporting a benefit. The last two conditions, especially Condition 4, did not.

As a whole, the results from the first two conditions indicate that providing the Experimental Model with a single bit of information about the training samples modality improves performance. In Condition 1, an accuracy improvement occurred in both training and validation tasks, and a reduced number of epochs were required to reach this accuracy. In Condition 2, this improvement manifested as a decrease in the training epochs that were required. Additionally, Condition 1's control models performed on par with the Baseline Model, meaning that the Experimental Model's performance was unlikely to have been due to the increase in trainable parameters required to incorporate the Hebbian learner's output into that model.

The results of Condition 4 are contrary to those of the first two conditions. Providing the Experimental Model with additional information significantly degraded the models average classification performance. These results are not entirely problematic for the findings of Conditions 1 and 2. Firstly, like Condition 1 control performance, this result decreases the likelihood that the significant differences obtained in the first two conditions were due to increased trainable parameters in the model; increasing the number of parameters led to decreased performance during Condition 4. Secondly, these results may indicate an interaction between the model architecture and the trained dataset. The only way to resolve this second issue is to repeat the experiment with many more neural network architectures, which was not possible here due to resource constraints.

### 4.3 Phenomenal consciousness in artificial intelligence

We designed the present experiment to emulate P-Consciousness's phenomenological property to determine whether limited forms of phenomenology provides any utility to an AI model and, by extension, the possible utility for that property within organic beings. Our emulation consisted of providing the Experimental Models with the equivalent of one bit of information (1 or -1 in this case) that represented whether the current training sample was predicted to have been a sound or an image file. That representation provided the Experimental Models with a way to separate the modalities while learning classification tasks. By analogy, a human may not be able to identify a new object or sound but is aware that the visual system experiences objects and that the auditory system experiences sound but not *vice versa.*

In effect, our experiment treated phenomenology as a shortcut to important information. This shortcut is similar to an idea raised by Zaadnoordijk and Besold (2018) when discussing implementations of phenomenal experience in AI. They suggested that phenomenal experiences can be viewed as a direct mapping from sensory inputs to mental representations that bypass many high-level cognitive functions and provide information more efficiently than when it needs to be fully processed. They also argued that systems with P-Consciousness-like properties should implement the phenomenal systems as a parallel process rather than embed it within the primary system. Our experiment is consistent with their proposal as the pre-trained model and attached Hebbian learner run parallel to parts of the neural network being trained.

Others have approached synthetic phenomenology by attempting to create qualia (basic units of experience; Loosemore, 2009; Schwitzgebel, 2016) within their systems (see Arrabales et al., 2010b; 2011). While this is a valid strategy that differs from our own, it highlights an issue that occurs when contributing to this field. As Arrabales et al. (2011) point out, there is no generally accepted model for qualia in biological beings; therefore, it is difficult to model them. Others have raised similar issues (e.g. Gamez, 2019). With this in mind, it is of interest to see whether the single bit of experiential information of our experiment shares any properties with qualia models. As we do not have a working memory component to our model, we can rule out any qualia model such as Arrabales et al.'s that depends on the Global Workspace Theory (Baars, 1988).

We could make the argument that the information provided by the Hebbian learning in our model is consistent with a quale from Ramachandran and Hirstein (1997) three laws of qualia. The first law is satisfied because the model cannot decide to experience the information differently, making it irrevocable. We did not force the neural network to produce the same output during the experience, which satisfies the 2^nd^ law. The main issue is the 3^rd^ law that requires that the experiential information exist long enough to be experienced, which Ramachandran and Hirstein suggested requires working memory. Although we, as with all other works in synthetic phenomenology, do not claim that the AI models are experiencing anything, perhaps the momentary existence of the experiential information in our model is long enough to be consistent with Ramachandran and Hirstein's model.

It is also challenging to determine whether the Hebbian learner's output can also be considered consistent with qualia under Information Integration Theory (IIT; Tononi, 2004). On the one hand, the output is maximally irreducible, as Oizumi et al. (2014) described, as it cannot be separated into sub-experiences. On the other hand, Balduzzi and Tononi's (2009) describe qualia as having a complicated geometric structure arising from binary operations. IIT is typically described using binary switches, so it is not easy to apply IIT directly to neural networks using continuous variables.

Geometric structures also appear in other models of consciousness that could be of relevance to our experiment. Recently, models of consciousness based on quantum decision making have been proposed that incorporate context into the equation (Ishwarya & Cherukuri, 2020a, 2020b). According to these models, consciousness arises due to high-dimensional representations of concepts within a contextual sub-space. Information within the hidden layers of a DNN are already high-dimensional representations of data, so we could argue that providing a modality label to neural networks changes the context within that representation. These added changes should then influence the model's decision making (i.e. output), which was reflected in our results.

### 4.4 Applications of experimental models

One limitation of the present experiment was that we had to train the Hebbian learners had using supervised learning. Therefore, it would be not easy to apply this method to unsupervised settings directly. One potential method could be to use something like DeepCluster (Caron et al., 2018) that creates classes while training. Instead of clustering the output, we could cluster information within the hidden layer to create the classes required to train the Hebbian Learner.

### 4.5 Conclusion

The present experiment was an attempt at two different tasks. The first was to extract simple representations of the modality being processed by the DNN using information from the hidden layers within that network. The second task was to determine whether feeding these representations into a new DNN lead to improved network performance.

We were successful with the first task as representations could be recovered from the network in most experimental conditions. We also observed that our accuracy in recovering these representations changed throughout an experiment.

We obtained mixed results during our second task. When the DNN was optimised to learn a particular dataset, the representation led to improved network performance in validation accuracy and decreased the amount of training required. When the DNN was not optimised for the dataset, the performance was either negatively impacted or unaffected.

While more work is required before we can determine the efficacy of our approach to improving DNN performance, our results do show some promise. It would also be of interest to see how far we could push our model's phenomenological aspects. For example, could future models extract colours or pitch from the hidden layers, and if so, would that cause further improvements to the model. For now, all we can say is that the ability to separate sounds from images can lead to some improvement in performance.

## 5 Limitations of the study

This study opted for a between-groups experimental design that ran large models multiple times to create group averages. Due to this, we only tested a few neural network architectures. Future studies could investigate a broader range of architectures to test the robustness of the effect described here. Additionally, we trained the Hebbian learners using supervised learning where the samples' origin was already known. Additional techniques would need to be developed to apply this model to an unsupervised learning setting.

## Declarations

### Author contribution statement

J. L. Bensemann: Conceived and designed the experiments; Performed the experiments; Analyzed and interpreted the data; Contributed reagents, materials, analysis tools or data; Wrote the paper.

M. Witbrock: Conceived and designed the experiments; Analyzed and interpreted the data; Contributed reagents, materials, analysis tools or data.

### Funding statement

This research did not receive any specific grant from funding agencies in the public, commercial, or not-for-profit sectors.

### Data availability statement

Data will be made available on request.

### Declaration of interests statement

The authors declare no conflict of interest.

### Additional information

No additional information is available for this paper.

## Appendix A Supplementary data

Supplemental Information submission 5 Supplemental Information submission 5
