# Supplementary material for: The effects of implementing phenomenology in a deep neural network
Source: Heliyon. 2021 Jun 8;7(6):e07246. doi: 10.1016/j.heliyon.2021.e07246 (PMC8214092; doi:10.1016/j.heliyon.2021.e07246)

## Supplemental Information

## Figure Captions

Figure S1: Condition 1 Training Results: The average training loss, training accuracy, validation loss, and validation accuracy of all four models trained during Condition 1 (Entity task with optimised models).

Figure S2: Condition 1 Training Results: The average training loss, training accuracy, validation loss, and validation accuracy of all four models trained during Condition 1 (Entity task with optimised models). Data are presented with 95% confidence intervals.

## Supplementary Figures

### Figure S1

###
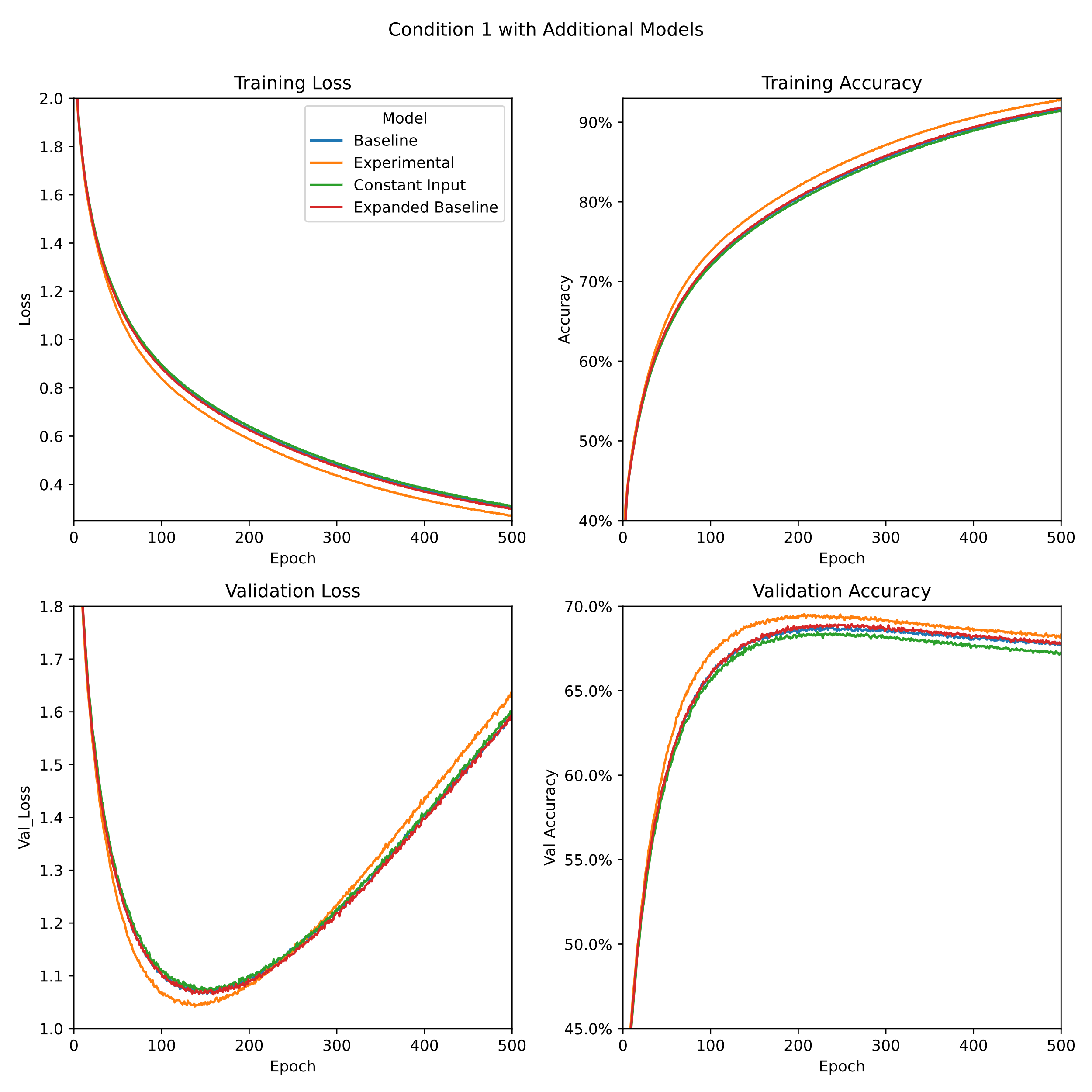


### Figure S2

###
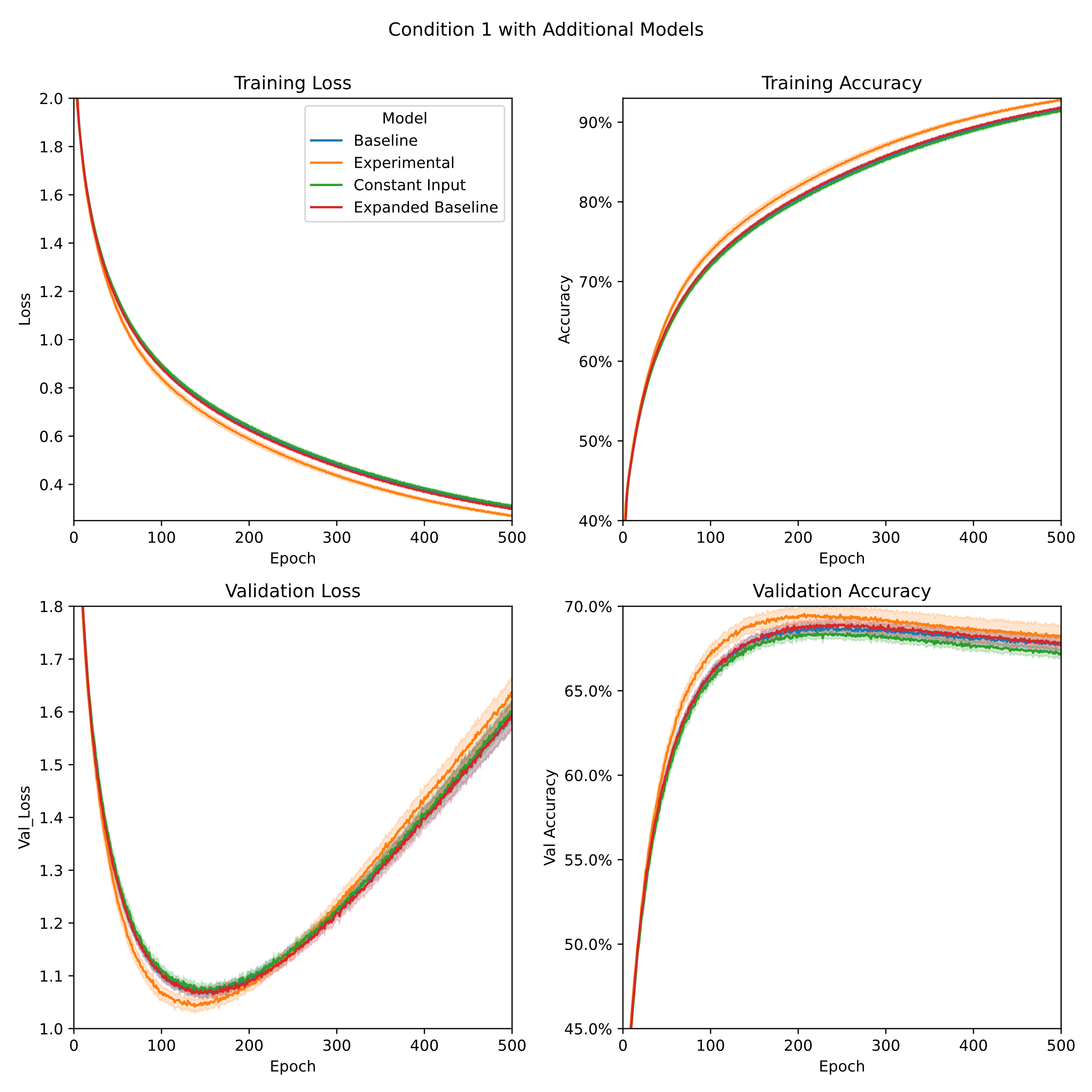

Supplement: Supplemental Information submission 5 [file mmc1.docx]
